# Supplementary material for: Learning few-shot imitation as cultural transmission
Source: Nat Commun. 2023 Nov 28;14:7536. doi: 10.1038/s41467-023-42875-2 (PMC10684502; doi:10.1038/s41467-023-42875-2)
Supplement: Supplementary file 1 — Supplementary Information [file 41467_2023_42875_MOESM1_ESM.pdf]

Learning Few-Shot Imitation as Cultural Transmission: Supplementary Information

A Glossary

Here we provide a glossary with definitions of key terms, for the reader's convenience.

A *policy* for an agent is a distribution over actions for each state in the environment. In our setting, the actions that can be taken are physical movement, so a policy represents a physical movement behaviour.

Cultural transmission from expert to agent is defined to be the extent to which the agent can improve its score during and after the expert has been present in the world. See Section 2.2 for a mathematical formalisation of this.

Imitation is the ability of an agent to copy salient features of the policy of a third-person demonstrator in real time. This is known as few-shot, embodied, third-person imitation in the machine learning literature. It is a particular (high-fidelity) kind of cultural transmission.

Adaptation is the ability of an agent to improve its policy online, on-the-fly and from a single stream of experience based on the discovery of new information. In our setting, adaptation is typically made possible by an agent's internal memory.

Social learning is the process by which cultural transmission occurs; i.e., an individual's acquisition of previously unknown information or behaviour by observing another individual in real time. It is a kind of adaptation facilitated by the presence of a co-player in the environment.

The *fidelity* of cultural transmission is the extent to which the (score-salient) behaviour of the expert can be reproduced by the agent without mistakes. In our setting, this corresponds to the agent reliably reproducing the same correct patterns of sphere visits as the expert.

Generalisation is an agent's ability to successfully demonstrate cultural transmission across a wide variety of different environmental conditions, including in environment variants not seen during training.

Recall is the ability of an agent to reproduce the (score-salient) behaviour of an expert after that expert has departed from the world.

The *robustness* of a policy is a shorthand for referring to the policy's generalisation, fidelity and recall abilities.

A *probe task* is a hand-picked evaluation task which is (probabilistically) held-out from training, and used to assess the robustness of cultural transmission. Probe tasks are held fixed

so we can get a fair comparison across agents ablated in different ways.

Expert dropout is a training procedure in which each episode of experience for the agent consists of some period(s) in which the expert is present and some period(s) in which the expert is absent ("dropped out").

Automatic domain randomization is a training procedure in which each episode of experience for an agent consists of a task sampled from a distribution, the parameters of which gradually change over the course of training, based on the value of a metric (in our case, cultural transmission).

B Details of GoalCycle3D Environment

B.1 World Space

Each world is built using the Unity game engine [48, 91]. It is a three-dimensional simulated physical space in which avatars are situated. An avatar is an embodiment of a player in the virtual world and is capable of perception and movement. Each world is perfectly square, of size between $16 \times 16\text{m}^2$ and $37 \times 37\text{m}^2$. The bumpy terrain is procedurally generated using Libnoise [11], parameterised by frequency and amplitude. The playable terrain is bounded by an impermeable barrier, invisible to players but visible to human observers.

Within the playable terrain are procedurally-generated horizontal and vertical obstacles, parameterised by density. The obstacles create navigational and perception challenges for players. In empty worlds, the path between two goals is often immediately visible, and typically a straight line. In worlds with obstacles, visibility can be highly restricted, since obstacles block vision and the LIDAR sensors used by our agents (see Section B.3). Moreover, players may need to take complex paths to reach a goal, including jumping or crouching, requiring continued actions for many steps. Supplementary Figures 1a and 1b illustrate some representative worlds using different parameters and seeds.

Vertical obstacles are perpendicular to the terrain and require the player to move around them. Their density can reach up to $0.05\,\text{m}^{-2}$. In a flat terrain of size $24\,\text{m}^2$, the maximum value produces 29 vertical obstacles on average. *Horizontal obstacles* lie flat on the terrain and require the player to jump over or (in rarer cases) crouch under them. Their density can reach up to $0.007\,\text{m}^{-2}$. In a flat terrain of size $24\,\text{m}^2$, the maximum value produces 4 horizontal obstacles on average.

Obstacles are constrained to spawn only on surfaces with a absolute value of slope $< 10°$ for vertical obstacles and $< 30°$ for horizontal obstacles. Therefore, given a fixed obstacle density, there is an inverse relationship between the bumpiness of the terrain and the density of the obstacles. Each horizontal obstacle is of fixed length 16 m and random rotation in the horizontal plane. Each vertical obstacle is of diameter sampled uniformly at random between 0.75 m and 2.25 m, and of sufficient height that no player can jump over it.

B.2 Game Space

Players receive a reward of +1 for entering a goal in the correct order, given the previous goals entered. The first goal entered in an episode always confers a reward of +1. If a player enters an incorrect goal, they receive a reward of −1 and must now continue as if this were the first goal they had entered. If a player re-enters the last goal they left, they receive a reward of 0. The optimal policy is to divine a correct order, by experimentation or observation of an expert, and then visit the spheres in this cyclic order for the rest of the episode. At the start of each episode, goals are placed randomly in the world, subject to the constraint that they do not

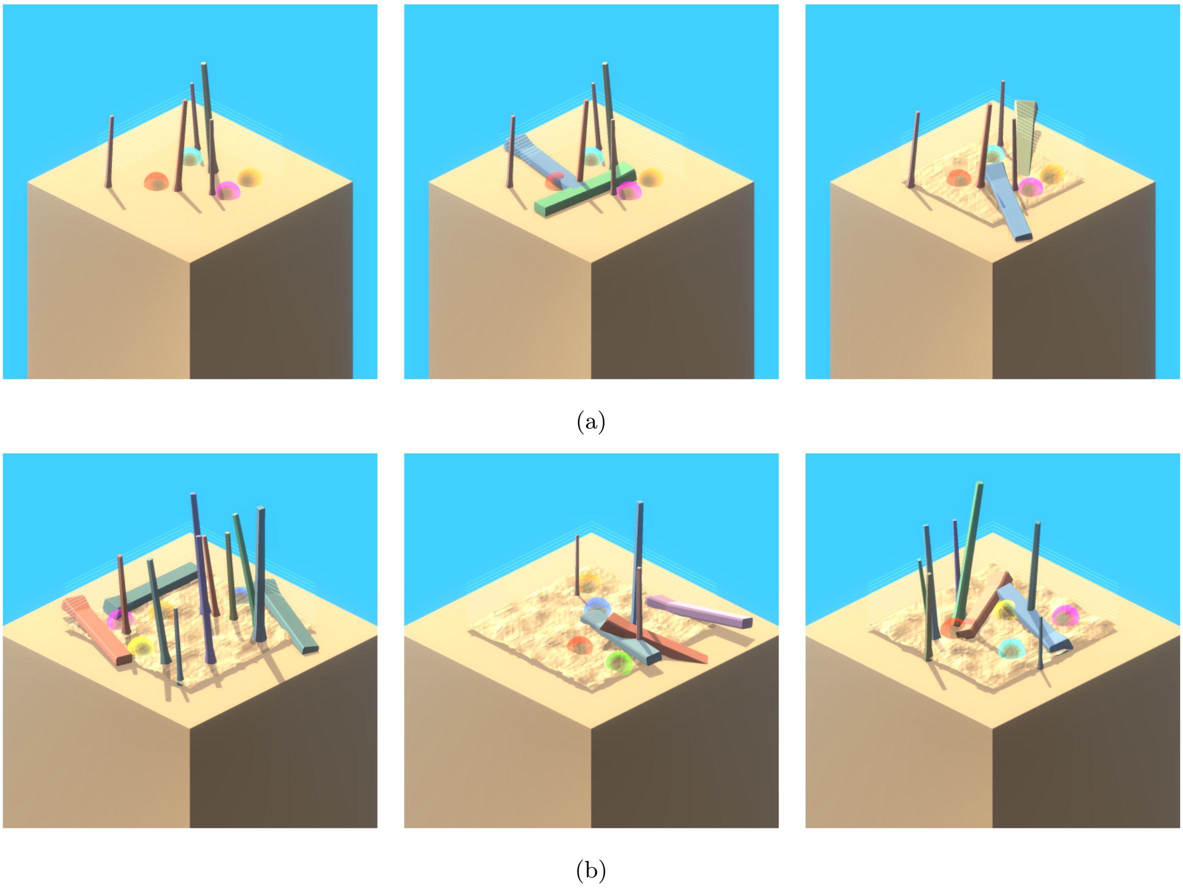

Supplementary Figure 1: Example procedurally-generated worlds with varying parameters and seeds. (a) Three worlds created from the same seed but with different procedural generation parameters. (b) Three worlds generated from the same procedural generation parameters but different random seeds.

overlap, achieved via rejection sampling. Supplementary Figure 2 illustrates some of the game mechanics.

Players are positively rewarded for visiting *goal* spheres in particular cyclic orders. In each possible order, every goal appears exactly once. Therefore, the set of distinct orders may be conveniently expressed as cyclic permutations of maximum length. To construct a game, given a number of goals n, an order σ is sampled uniformly at random. The positively rewarding orders for the game are then fixed to be $\{\sigma, \sigma^{-1}\}$ where σ^{-1} is the opposite direction of the order σ. An agent has a chance $\frac{2}{(n-1)!}$ of selecting a correct order at random at the start of each episode. In all our training and evaluation we use $n \geq 4$, so one is always more likely to guess incorrectly. Note that σ and σ^{-1} are equally difficult, equally rewarding options, provided that the world is not chiral. That is to say, assuming there is no difference between the world and its mirror image from above with respect to performing a given trajectory.

We can classify paths between goals according to their topology. Distinct topologies are characterised by the number of self-intersections on the path, which we refer to as "crossings". Different topologies present players with different challenges, since the relative position of the next goal with respect to the others is altered. Moreover, topologies with a higher number of crossings tend to have longer paths. During training, we use rejection sampling to achieve a uniform distribution over topologies. Supplementary Figure 3 depicts all possible topologies in games with 4, 5 and 6 goals, which we refer to as 4-, 5-, and 6-cycle tasks.

In mathematical terms, the distinct orders are Hamiltonian cycles on the complete graph K_n where n is the number of goals, the nodes of the graph are the goals, and the edges represent equivalence classes of paths between one goal and another, without passing through any inter-

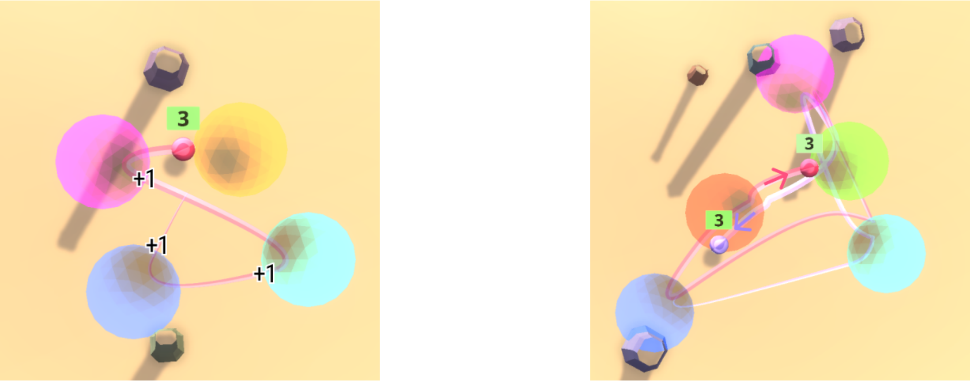

(a) (b)

Supplementary Figure 2: Illustration of game mechanics. (a) A single player navigates between four goals, with annotations showing when the score changes. (b) Two players demonstrate the two equally rewarding paths available between five goals.

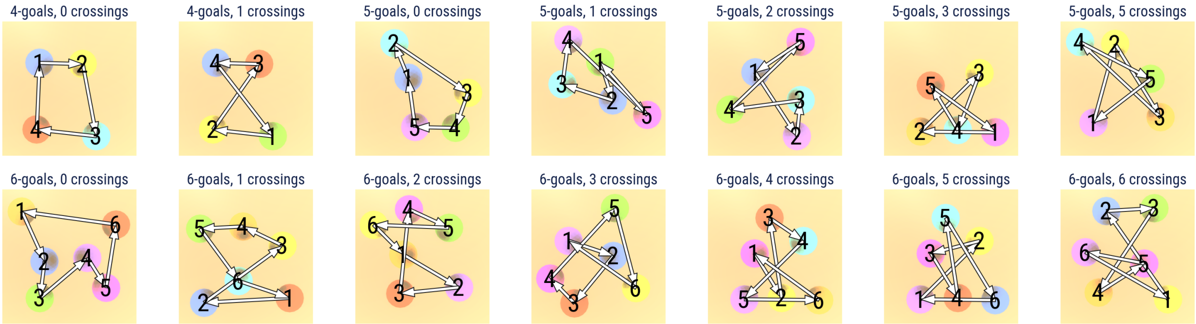

Supplementary Figure 3: An overview of all 4- 5-, and 6-cycle topologies, characterised by the number of crossings in their shortest paths.

mediate goal. In K_n there are $(n-1)!$ distinct Hamiltonian cycles, as may be readily verified by a counting argument. More formally, the order σ^{-1} is the group inverse of σ when viewed as an element of the symmetric group S_n.

In principle, subcycles may also be positively rewarding, but we show empirically that these are not optimal on average in Supplementary Table 1. Goal sphere colours are sampled uniformly at random without replacement from a set of 8 possibilities, depicted in Figure 1 (bottom left). The diameter of each goal sphere is fixed as a function of the world size w to be $\frac{w}{8}+2$. Note that sampling the rewarding cycle and goal positions uniformly at random does not produce a uniform distribution over topologies.

B.3 Player Interface

Players are embodied as physical avatars (Supplementary Figure 4). Avatars interact with the environment simulation on discrete timesteps. On each timestep an avatar perceives its surroundings using sensors and converts this to an observation. This observation is sent to the player who must return an action vector, which the avatar converts to actuation values passed to the environment simulator. The environment simulator takes actuation information from all avatars and performs a step of physics simulation to generate the next global state for the avatars to perceive. The avatar is surrounded by a collision mesh, which can be disabled to allow evaluation of agents alongside pre-recorded human trajectories.

The action space is 5-dimensional and continuous, with each action dimension taking values in $[-1, 1]$. The five dimensions represent moving forwards and backwards, moving left and right,

Length of subcycle	Average score
2	1.2 ± 1
3	8.6 ± 1
**4**	$\mathbf{20.4 \pm 2}$

(a)

Length of subcycle	Average score
2	1.2 ± 1
3	8.3 ± 2
4	12.6 ± 2
**5**	$\mathbf{23.5 \pm 3}$

(b)

Supplementary Table 1: Average scores for subcycles of a representative (a) 4-goal game and (b) 5-goal game. The subcycles were sampled such that each goal sphere correctly follows the preceding sphere in one of the correct orders of the full cycle, with a disconnect only allowed at the beginning and end of the subcycle. Each row represents the average and one standard deviation of the score over 20 samples obtained by an expert bot following a subcycle of the given length. All the tasks used a 32×32 world with flat terrain and no obstacles.

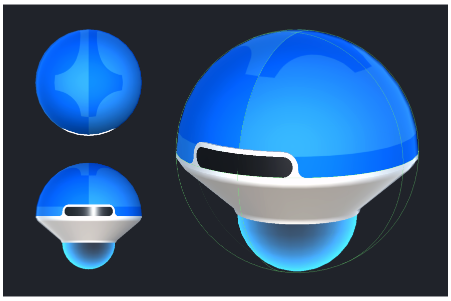

(a)

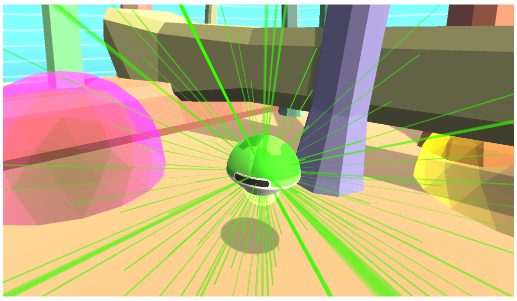

(b)

Supplementary Figure 4: Players are embodied as physical avatars. (a) Avatars are surrounded by a spherical collision mesh, shown in the left in green. (b) Agents perceive the world through LIDAR. LIDAR sensor rays emanate from the avatar's centre (shown here are rays which collide with objects). See Supplementary Movie 30.

rotating left and right, rotating up and down, and jumping and crouching. Players may take any combination of actions simultaneously. Humans interact using a discretised action set, with each action having values in $\{-1, 0, +1\}$, mapped to keyboard inputs. Movement dynamics are subject to inertia: players continue to move in their current direction, albeit at a diminishing rate, even when not sending any movement actions corresponding to that same direction.

Avatars can be controlled by a Unity scripted player, which we dub an *expert bot*. Expert bots are "oracles", receiving privileged information about the correct order of goals to traverse, navigating using the Unity NavMesh [86], and jumping and crouching when colliding with horizontal obstacles. These movement patterns are simple heuristics, so the expert bots are not guaranteed to find the most efficient trajectory from one goal to the next.

Human players observe the environment through an egocentric first-person camera with a resolution of 640×480 pixels. Agents observe the environment through a LIDAR sensor. The LIDAR sensor performs raycasts uniformly distributed in polar coordinates, with azimuth ranging from $0°$ to $360°$, altitude ranging from $-90°$ to $+90°$, and a grid of 14×14 rays. Each ray returns a one-hot encoding of the object with which the ray has intersected (vertical obstacle, horizontal obstacle, goal, avatar, terrain), its distance and, for goals only, its RGB color. The ray only returns the first object with which it collides. Therefore, for agents, all objects are opaque, including goals, and the boundary of the world is invisible. In addition, each agent is equipped with an AVATAR sensor, which outputs the 3-dimensional relative distance of the nearest co-player in Cartesian coordinates in the frame of reference of the avatar. This is used as a regression target during training but not passed as an input to the agent's neural network, so is not required at test time. Supplementary Figure 5 compares the human RGB and agent LIDAR observations.

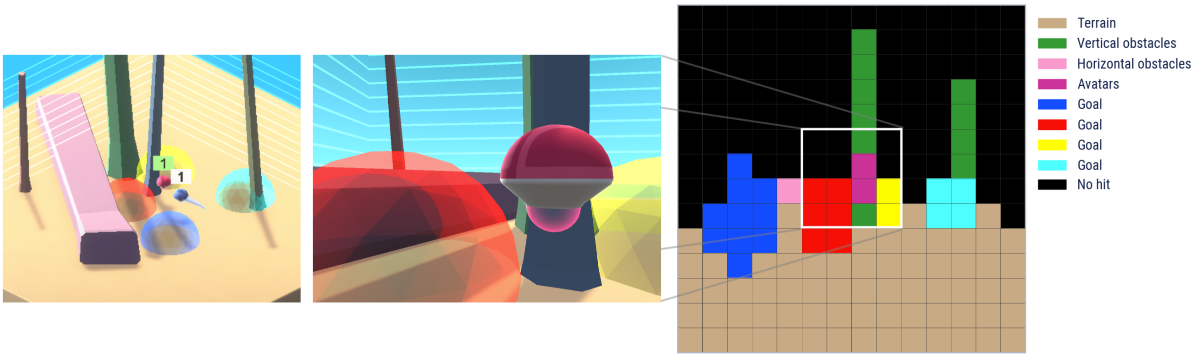

Supplementary Figure 5: (left) A third-person camera view at an instant in time. (centre) The human player RGB observation for the blue avatar. (right) A visualisation of the agent LIDAR observation for the same avatar. Each ray also reports the distance to the hit point, which is not shown in this rendering. The human player observation corresponds approximately to the highlighted central 4×4 array of the agent LIDAR observation.

Viewing distance for both humans and agents is fixed at 128 m, sufficient to perceive to the edge of the world from anywhere in the world for all world sizes used in this work. For humans, obstacles, terrain and avatars are opaque, goals are translucent and the boundary of the world is visible. Humans also perceive a box floating above each avatar displaying the avatar's current score, which is reset to 0 at the beginning of each episode. LIDAR is a common and efficient observation modality for real-world robots (e.g. [60]) and in prior multi-agent 3D physical simulated worlds (e.g. [6]). For agent LIDAR, the zero direction for the azimuth is fixed to be the FORWARD direction for the avatar, so rotation of the avatar is meaningful perceptually.

B.4 Probe Tasks

While our training and evaluation probe tasks are sampled from the same procedural generator, we use different random seeds for training and evaluation to ensure these probe tasks are held out, following [46, 61, 97]. When using ADR, the vast majority of training tasks have different parameters to our probe tasks and are therefore different by definition. For identical task parameters, the hold-out is probabilistic in nature. Using a worst-case argument for 5-goal games (our best agents were never trained on 4-goal games), suppose we placed goals on a discrete grid with blocks of size $6\,\text{m} \times 6\,\text{m}$ (corresponding to the goal sphere radius in $32\,\text{m} \times 32\,\text{m}$ worlds) thereby ensuring that overlaps are impossible. This would result in $\binom{25}{5} = 53130$ possible goal placements. We also sample 5 out of 8 colours, giving 3×10^6 possible goal positions and colours. For flat, empty worlds of size $32\,\text{m} \times 32\,\text{m}$ we only ever sample a maximum of 50 such goal placements and colours during training and 12 during evaluation using different seeds. It is therefore highly improbable that any single training task is the same as a probe task.

For the complex tasks with obstacles, consider a grid with blocks of size $1\,\text{m} \times 1\,\text{m}$ and an average of 3 vertical obstacles and 3 horizontal obstacles. There are $\binom{1024}{3} = 1.8 \times 10^8$ possible placements for each of these obstacle types (note that obstacle overlaps are allowed by our procedural generator). Multiplying the number of goal placements and colours by the number of vertical obstacle placements and horizontal obstacle placements gives 9×10^{22} possible tasks. Again in complex tasks we sample 50 possible world seeds which control the placement of these obstacles, which combined with 50 goal seeds gives only 2500 possible training tasks with the same task parameters used in our probe tasks. The likelihood of these training tasks overlapping the 12 probe tasks we sample is vanishingly small. Note that this already conservative argument ignores the random rotation of horizontal obstacles and also ignores the terrain bumpiness and therefore significantly undercounts the number of possible tasks in practice.

C Supplementary methods

C.1 Reinforcement Learning Formalism

Let $\mathcal{D}(\Omega)$ denote the space of distributions over the space Ω. A Markov Decision Process (MDP) [42, 83] is a tuple $\langle \mathcal{S}, \mathcal{A}, T, r, \gamma \rangle$ where $\mathcal{S}$ is a set of states, $\mathcal{A}$ is a set of actions, $T : \mathcal{S} \times \mathcal{A} \rightarrow \mathcal{D}(\mathcal{S})$ is a transition function, $r : \mathcal{S} \times \mathcal{A} \rightarrow \mathbb{R}$ is a reward function, and $\gamma \in [0, 1]$ is a discount factor. A mapping $\pi : \mathcal{S} \rightarrow \mathcal{D}(\mathcal{A})$ is called a stochastic policy. Given a policy π and an initial state s_0, we define the value function $V_\pi(s_0) = \mathbb{E}[\sum_{t=0}^{\infty} \gamma^t \; r(s_t, \pi(s_t))]$ where s_t is a random variable defined by the recurrence relation $s_t = T(s_{t-1}, \pi(s_{t-1}))$. Reinforcement learning seeks to find an optimal policy π^* which maximises the value function from an initial state s_0. We assume that the agent experiences the world in episodes of finite length T. During training, our RL agent receives many episodes of experience, and updates its policy to become closer to the optimal policy, as measured on a held-out episode during testing.

A partially-observable Markov Decision Process (POMDP) is defined by the tuple $\langle \mathcal{O}, \mathcal{A}, T, r, \gamma \rangle$, where each element of $\mathcal{O}$ is a partial observation of a true underlying state in $\mathcal{S}$. Typically, multi-agent settings are automatically POMDPs because each agent does not have access to the observations, actions, policies or rewards of their co-players [57]. This is especially true in our case, since co-players may also drop in and drop out of the environment within the course of an episode (see Section 4.3).

A crucial challenge in RL is the balance between exploration and exploitation. Exploration involves discovering new parts of the state-action space and their implications for value; exploitation involves using learned information about valuable states and actions to gain reward [49]. Exploration is challenging when reward is sparse or deceptive and the state-action space is large. There may be many steps required to discover an improved strategy which forego the reward offered by exploitation. In such "hard exploration" problems, RL is prone to falling into local optima. We show how an independent agent can learn to model its environment, and particularly others therein, to solve held-out hard exploration problems.

Reinforcement learning of cultural transmission cannot occur in fully-observed tabular settings where the environment reward is unaffected by the behaviour of the expert. The proof of this is straightforward. For an agent to learn cultural transmission, they must be rewarded in states where the behaviour of an expert co-player is salient. However, in a fully observed setting, these states also contain perfect information about the environmental features. By assumption these features determine the agent's reward independently of the behaviour of the expert. If the setting is tabular, there is no aliasing of states, so the behaviour of the expert is irrelevant from the perspective of the Q-function.

In our setting we violate the assumptions of this lemma in two ways: by operating in a partially observable setting, and by operating in a rich 3D physical world that necessitates function approximation. Under these conditions, with appropriate domain randomisation and attention biases, we show that reinforcement learning on environment reward alone is sufficient to learn a cultural transmission policy. The corresponding neural network aliases states in such a way that it can both infer hidden information from the expert and reuse that information after the expert has dropped out.

Note that the optimal policy π^* in an environment containing other agents is at least as good as the optimal policy in the same environment without other agents. This is true because the set of policies in the augmented world is a superset of the policies in the non-augmented world, by virtue of the richer state space in the augmented world. Practically speaking, an agent should be able to leverage behaviour of other agents when beneficial without regressing solo performance.

C.2 Maximum a Posteriori Policy Optimization (MPO)

Policy improvement uses Expectation Maximisation (EM) coordinate ascent, alternating between a non-parametric E-step and a parametric M-step. The E-step re-weights state-action samples, assigning them weights q_{ij} according to Equation 6. Here a_i refers to an action sampled under state s_j, Q^π is the Q-function of the current policy $\pi^{(k)}$, Z_j is a normalisation term and the temperature η is a Lagrange multiplier. The M-step solves the primal optimisation problem in Equation 7 by performing one iteration of optimising the Lagrange multiplier α holding the policy parameters θ constant, and then optimising θ holding α constant. The KL regularisation term prevents the policy from changing "too quickly" based on samples from a potentially inaccurate Q-function. This regularisation term is averaged over the K states sampled and its strength is controlled by the hyperparameter ϵ. Retrace ([67]) is used for policy estimation using off-policy trajectories. When used in continuous control, MPO fits the mean and covariance of a Gaussian distribution over the joint action space.

$$q_{ij} = q(a_i, s_j) = Z_j^{-1} \exp\left(\frac{Q^\pi(s_j, a_i)}{\eta}\right). \quad (6)$$

$$\max_\theta \min_{\alpha>0} L(\theta, \eta) = \max_\theta \min_{\alpha>0} \left[\sum_j \sum_i q_{ij} \log \pi_\theta(a_i|s_j) + \alpha\left(\epsilon - K^{-1} \sum_j \mathrm{KL}\left(\pi^{(k)}(a|s_j) \,||\, \pi_\theta(a|s_j)\right)\right)\right]. \quad (7)$$

C.3 Training Framework

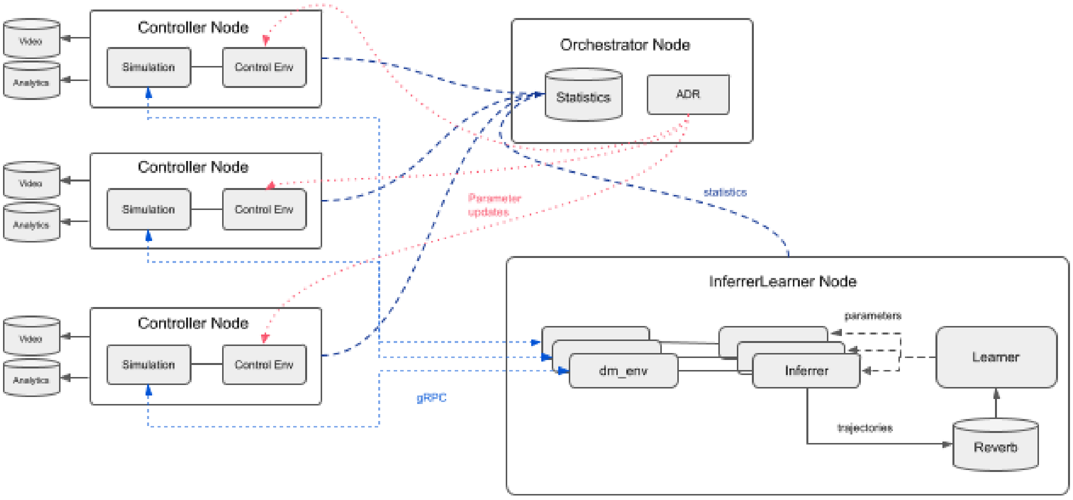

Supplementary Figure 6: High level system architecture diagram, showing the relationship between *InferrerLearner*, *World* and *Orchestrator* nodes.

Our distributed training architecture is depicted in Supplementary Figure 6. Each experiment has 192 independent *GoalCycle3D* simulations, capable of accepting connections from any number of players (agent or human). These simulations run on Nvidia Tesla P4 and V100 GPUs, with 8 simulations per GPU. Connected to each environment is a *(World) Controller* node which connects to the simulation using an environment interface. The environment interface collects analytical data from the simulation, including raw RGB observations from a

third-person camera that are stitched together and periodically saved as videos. The *Controller* node also manages episodes by keeping track of the (synchronised) steps taken by connected players and indicating the end of an episode to the simulation after 1800 steps have occurred. The simulation handles synchronisation by blocking until all connected players have sent their action before stepping. It also signals the end of an episode to connected players by setting a "last step" flag in the connected player's observations. The *Controller* also manages "soft resetting" of the simulation, where simulation parameters such as number and position of goals, vertical and horizontal and obstacle densities, terrain bumpiness, avatar spawn positions and world size are updated before re-rendering the simulation. These soft resets are aligned with the synchronised episode boundaries.

Each agent in an experiment is controlled by an independent *InferrerLearner* node. These nodes do not communicate with each other. Each *InferrerLearner* node connects to all of the running simulations and communicates with these (sending actions and receiving observations) using a remote `dm_env` ([66]) interface over gRPC. Thus, even though this is a multi-agent architecture, the problem looks like a local, single agent RL problem to an individual agent. Each *InferrerLearner* node is divided into an *Inferrer* and a *Learner*, which both run continuously, sharing a single Google Cloud TPU v2 host. This enables high speed memory access between the *Inferrer* and *Learner* for parameter synchronisation.

An *Inferrer* maintains a thread for each environment it is connected to which runs the standard RL loop of receiving an observation, passing this through the agent network to obtain an action and returning this action to the environment. Trajectories of observations, rewards, actions and additional metadata computed by the agent in all threads are saved to a single FIFO experience replay buffer implemented using Reverb ([23]).

The learner continuously queries the replay buffer for a batch of trajectories, blocking if insufficient data is available in the buffer. A copy of the agent's parameters are updated using gradient descent on this batch of trajectories, before pushing the updated parameters to the policies running on the *Inferrer* threads.

A final node, called the *Orchestrator* is responsible for coordinating the connection and disconnection of *InferrerLearner* nodes to and from simulations. The *Orchestrator* also keeps track of aggregate statistics from *Controller* nodes including total step counts and average scores, which are used to calculate a training CT metric. It coordinates simulation parameter updates through the ADR mechanism (see Section 4.5). These simulation parameter updates are pushed to the *Controller* nodes which store them in a buffer, waiting to apply to them to the simulation when the next episode boundary is reached.

The topology of the full distributed system, consisting of *Orchestrator*, *Controller*, *InferrerLearner* nodes and Reverb replay buffers is configured and mapped to hardware using Launchpad ([95]).

C.4 Training Hyperparameters

The agent receives observations from the environment, computed by LIDAR and AVATAR sensors described in Section B.3. The LIDAR sensor is used as an input to the agent's neural network, but the AVATAR sensor is not. We additionally feed the previous step's reward into the agent's neural network. The $14 \times 14 \times 9$ LIDAR sensor is passed through a convolutional layer with 24 square 2×2 kernels. The previous reward is concatenated to the output of this convolutional layer before being passed to a linear layer of size 128. Layer norm is applied before a *tanh* activation followed by 2 linear layers of size 256 with *elu* activation in between. The encoder is illustrated in Supplementary Figure 7, resulting in an encoded observation.

Supplementary Table 2 presents the hyperparameters used during training of our agents. The values were either the defaults found in the DeepMind JAX Ecosystem [4] or were set using small-scale sweeps early in experimentation.

Hyperparam	Description	Value
**MPO Hyperparameters** (see [1] for details)		
Policy samples	Number of actions to sample from the policy	100
Min stdev	A minimum (epsilon) standard deviation to add to the predicted value to prevent it from being 0.	1e-4
Action embedding	Transformation function applied to action samples	tanh
Alpha init	Initial value of Lagrange multiplier for KL mean and covariance	1
Epsilon alpha KL mean	Epsilon for Lagrange multiplier for KL mean	2.4e-2
Epsilon alpha KL cov	Epsilon for Lagrange multiplier for KL covariance	7e-5
Temperature init	Initial value of temperature Lagrange multiplier	0.325
Epsilon temperature	Epsilon for temperature Lagrange multiplier	2e-2
Gamma	Discount factor applied to returns	0.99387
**Training hyperparameters**		
Critic learning rate	Learning rate of ADAM optimiser [50] applied only to the critic neural network	4.8e-4
Actor learning rate	Learning rate of ADAM optimiser applied to policy, encoder, RNN and attention loss networks	2e-4
Network update period	Number of steps after which the online network is replaced with the target network	100
Attention loss weight	Relative weight of the attention loss relative to the MPO loss	10
Attention loss step	The (future) prediction step used by the attention loss	0
Batch size	The batch size for learner steps	32
Trajectory length	The number of steps over which the RNN is unrolled in each learner step	800
Episode length	The number of steps making up an episode. The agent's state is reset and it respawns at a random location at the beginning of an episode.	1800

Supplementary Table 2: Hyperparameters.

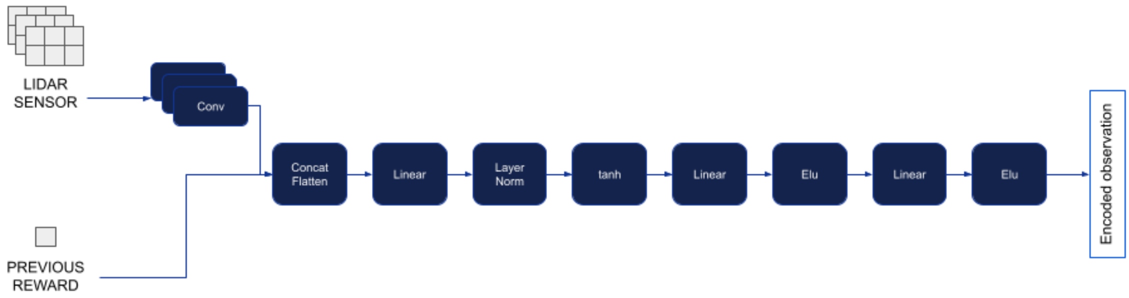

Supplementary Figure 7: Encoder architecture.

C.5 Automatic Domain Randomisation (ADR)

To train on a diverse set of tasks, the Orchestrator (see Appendix C.3) periodically re-samples all of the task parameters according to $\lambda \sim P_\phi$ and configures the environment simulations using the sampled parameters synchronously. Hence, between these resampling instances, the agent is trained on tasks distributed according to P_ϕ. Note that the resampling interval is set to be 10 minutes, much longer than the time it takes to complete the number of steps in an episode. In practice, 30 to 50 episodes are run using a task with the same parameters.

To adapt the task distribution to the agent's Goldilocks zone, the distribution parameters ϕ are updated before sampling task parameters in the Orchestrator. To facilitate this update, we follow two techniques from the original ADR: boundary sampling and threshold updates.

When sampling a parameter λ, with probability $p_b \in [0, 1]$ (referred to as the boundary sampling probability) a task parameter λ_b is chosen uniformly at random to be fixed to one of its "boundaries": $\{\phi_b^L, \phi_b^H\}$. In general, both boundaries are updated by ADR and the boundary value is randomly sampled from the two options. We also allow cases where only one of the boundaries is updated by ADR, while other is fixed (for example, if a parameter is non-negative). In these cases, the variable boundary is used as the sampled value. Once λ_b is determined, the remaining components of λ are sampled according to P_ϕ. Finally, with probability $1 - p_b$, λ is sampled directly from P_ϕ.

Each task parameter that was boundary sampled contains one component at index b that matches either ϕ_b^L or ϕ_b^H. Let $b(\lambda) \in \{1, \ldots, d\} \times \{L, H\}$ denote the mapping from a task parameter to the boundary to which λ_b was fixed, specified by a tuple: (b, L) or (b, H). At the end of an Orchestrator update interval, each simulation sends its training cultural transmission metric (see Section 2.2) to the queue $q(b(\lambda))$. Note that up to $2d$ separate queues are required. Simulations where λ was not boundary sampled do not push their cultural transmission metrics.

A training cultural transmission metric for a given set of task parameters is obtained by collecting agent scores in 3 special episodes with no dropout, full dropout, and half dropout of the expert bot. These episodes are run every time the Orchestrator resamples task parameters.

To update ϕ_i^L (and similarly ϕ_i^H), we simply average $q(i, L)$ and compare the average training cultural transmission metric $\bar{c}(i, L)$ against fixed thresholds: th_L, th_H. ϕ_i^L is updated according to

$$\phi_i^L = \begin{cases} \phi_i^L - \Delta_i & \bar{c}(i, L) > \text{th}_H \\ \phi_i^L + \Delta_i & \bar{c}(i, L) < \text{th}_L \\ \phi_i^L & \text{otherwise or q(i, L) empty} \end{cases}, \tag{8}$$

and ϕ_i^H according to

$$\phi_i^H = \begin{cases} \phi_i^H + \Delta_i & \bar{c}(i, H) > \text{th}_H \\ \phi_i^H - \Delta_i & \bar{c}(i, H) < \text{th}_L \\ \phi_i^H & \text{otherwise or q(i, H) empty} \end{cases}, \quad (9)$$

for Δ_i a fixed, positive step size.

Parameter	In-distribution range	Out-of-distribution ranges
World size	[20, 26]	[18, 19], [26, 31]
Horizontal obstacle density	[0.0001, 0.0024]	0.0, [0.0025, 0.003]
Vertical obstacle density	[0.0, 0.0255]	[0.026, 0.031]
Bumpy terrain max value	[0.0, 3.4]	[3.5, 4.1]
Bumpy terrain frequency	[0.01, 0.1]	[0.0, 0.008], [0.102, 0.12]
Bot speed	[9.0, 13.7]	[7.2, 8.6], [13.8, 16.4]
Probabilistic dropout transition probability	[2/1800, 40/1800]	0.0, [42/1800, 48/1800]

Supplementary Table 3: In-distribution and out-of-distribution parameter values for the evaluated MEDAL-ADR agent.

D Additional results

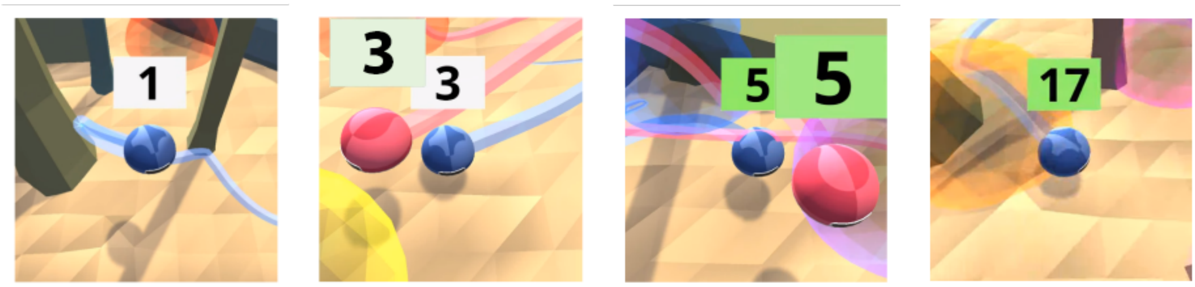

(a) (b) (c) (d)

Supplementary Figure 8: Freeze-frames from a single episode of test-time evaluation, in chronological order from left to right. (a) Our cultural transmission agent (blue avatar) is spawned in a held-out task; (b) the agent finds a human (red avatar); (c) the agent follows the human on a rewarding path through goals while navigating terrain and obstacles; (d) the agent recalls and reproduces the demonstrated path after the human has dropped out. The numbers above the avatars indicate cumulative score across the episode. See Supplementary Movie 31.

D.1 Training with Single-Parameter ADR

In this experiment, we use ADR to increase task complexity appropriately to maintain the Goldilocks zone for the learning of cultural transmission, controlling only the world size of the training tasks. Supplementary Figure 9 shows the training curves for the experiment.

The difference between Figure 2 and Supplementary Figure 9 is clear. In Supplementary Figure 9, training CT increases to near 1 and remains above 0.7 for the duration of the experiment without dropping towards 0. This shows the effect of applying ADR to a single parameter. At the start of training, before the world size upper boundary started to increase, all worlds are of size 16×16. This is the same as in the previous experiment, and we see the same progression in training CT through phases 1 to 3 as in Figure 2. However, as training CT increases above

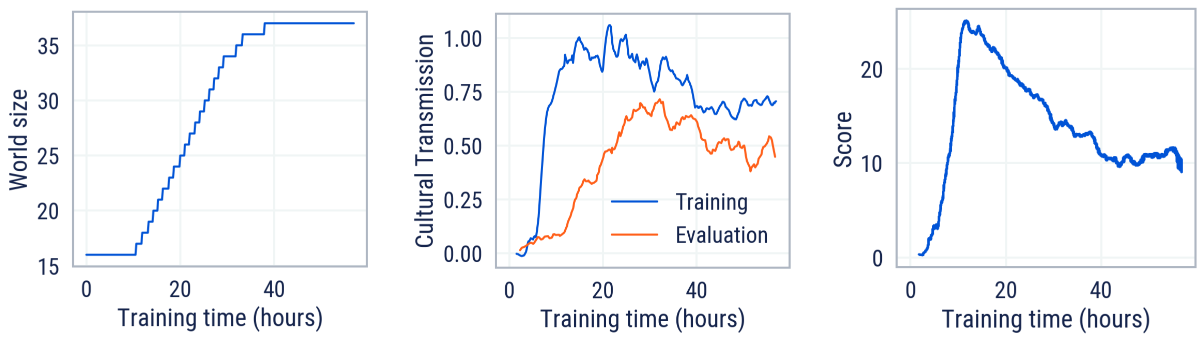

Supplementary Figure 9: Training curves for training with ADR controlling the world size parameter. (left) The upper boundary of the world size randomisation range, the lower boundary is fixed at 16. (centre) The cultural transmission metric as measured from training environments and held-out probe tasks. (right) The average avatar score over the training run. Note that the decreasing score does not indicate lower performance, since the maximum achievable score decreases with increasing world size.

a threshold (0.8 in this experiment), the upper boundary begins to increase in steps of 1. This continues until the maximum world size of 37×37 is reached. The progression of novel tasks prevents the collapse of CT towards 0: our agent no longer enters phase 4.

We highlight the advantage of using training CT to adapt boundaries in ADR rather than the agent score used in [69]. As a threshold-based method, ADR is sensitive to the scale and normalisation of the parameter used to adapt boundaries. As Supplementary Figure 9 shows, the CT is insensitive to changes in the maximum achievable score as the set of training tasks changes.

D.2 Training with Multi-Parameter ADR

In this experiment, we train our best cultural transmission agent by using ADR to control the set of task parameters in Supplementary Table 4. The parameters include changes to the world that make navigating the terrain more difficult (world size, bumpiness), additional skills to be learned (vertical and horizontal obstacles), and changes to the expert behaviour (bot speed, dropout transition probability). Together, these parameters interact to create tasks that increase in complexity and decrease in expert reliability.

Because the ADR parameter ranges can fluctuate over the duration of an ADR experiment, we use the following criteria to select the agent for analysis. First, we normalise the ADR parameter ranges at every training time step by the maximum range allowed according to Supplementary Table 4. The ranges are further averaged to obtain the mean ADR range. We also average the training cultural transmission metric of all ADR parameter boundaries at every training time step. Finally, we select a training time step that satisfies the following criteria:

- Mean normalised ADR range ≥ 0.55
- Mean training cultural transmission metric ≥ 0.80
- Min normalised ADR range ≥ 0.10

Supplementary Figure 10 shows the evaluation cultural transmission metric over time. Note that all probe tasks are strictly out-of-distribution: during training the world size never expands to 32×32, and human demonstrations are qualitatively different from an expert bot. Despite these differences, evaluation CT on empty 4-goal tasks reaches a final value of 0.85, indicating good generalisation and recall. In the 5-goal tasks, evaluation CT is lower. As we show in Section 2, our agent is still capable of recall and generalisation with 5 goals, but performance

Parameter	Min	Initial	Max	Step
World size	20	20	32	1
Horizontal obstacle density	0.0001	0.0001	0.01	0.0001
Vertical obstacle density	0.0	0.0	0.2	0.0005
Bumpy terrain max value	10.0	10.0	15.0	0.1
Bumpy terrain frequency	0.01	0.01	0.1	0.002
Bot speed	7.0	11.0	14.0	0.1
Probabilistic dropout transition probability	2/1800	20/1800	40/1800	2/1800

Supplementary Table 4: Task parameters controlled by ADR.

drops off in larger worlds. The prominent dip in evaluation cultural transmission metrics after 100 training hours is due to ADR starting to expand randomisation ranges at around 100 training hours. This leads to a momentary drop in social learning ability, which is amplified by the difficulty of evaluation tasks. ADR pauses the expansion until the agent recovers at around 150 hours.

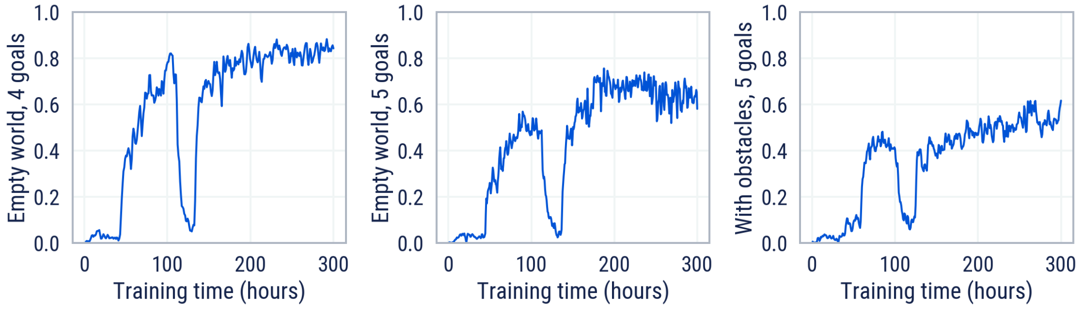

Supplementary Figure 10: Evaluation CT in three sets of probe tasks.

The process of emergent social learning with increasing task difficulty is gradual and the experiment run spanned more than 300 hours (1.5 weeks). To provide a sense of the experience consumed, the average rate of inference steps for the agent was approximately 4×10^6 per hour, and inference data was reused on average 55 times for learning. We note that all parameters controlled by ADR expanded significantly over the experiment run.

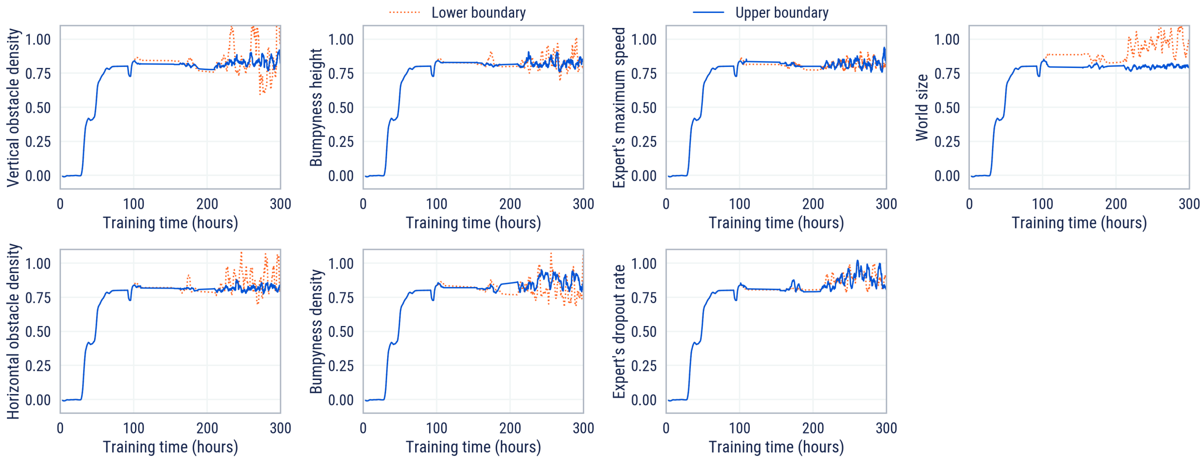

Supplementary Figure 11: Training CT metrics. ADR adapts each randomisation range boundary by expanding when the metric is above an update threshold high of 0.85 and contracting when the metric is below an update threshold low of 0.75.

Supplementary Figure 12 shows the agent and expert bot scores over the training run.

As before, when ADR expanded the parameter boundaries, the maximum achievable score (approximately equal to the bot score) decreased. The agent score also showed a corresponding decrease. The successful expansion of ADR reemphasises the importance of our choice to use the cultural transmission metric to adapt ADR, avoiding scale and normalisation issues with score. Finally, we observe the "spikes" in both bot and agent scores over the training run, likely due to expansions and contractions of world parameter randomisation ranges by ADR.

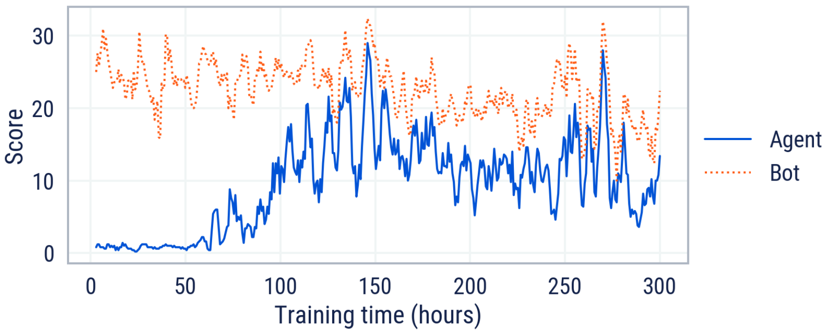

Supplementary Figure 12: Agent and bot scores over ADR training, smoothed using a moving average of length 5.

D.3 Introspecting the Agent's "Brain"

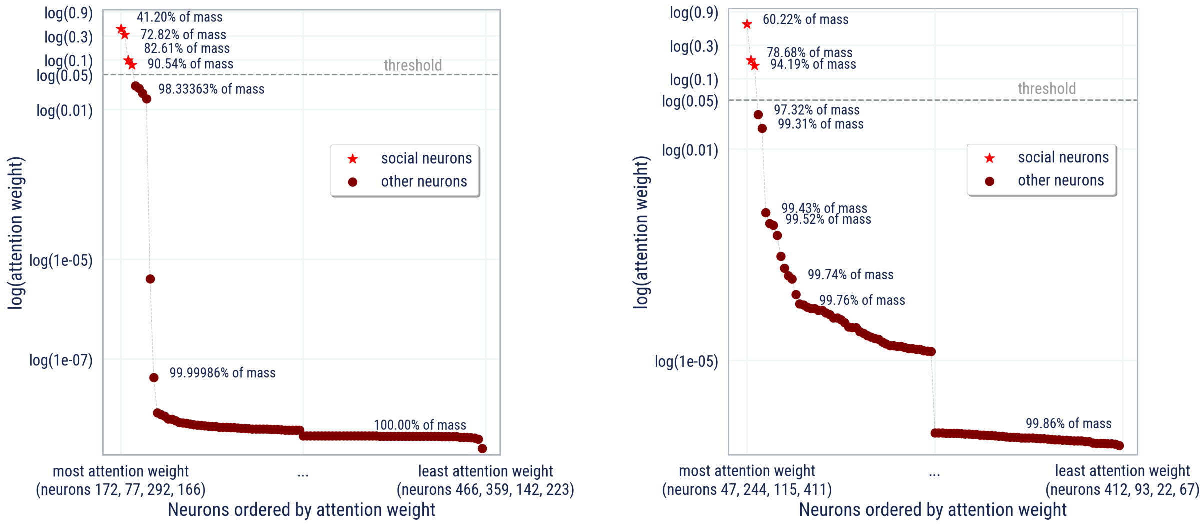

(a) (b)

Supplementary Figure 13: (a) MEDAL. (b) MEDAL-ADR. Ordering the neurons based on their corresponding (log) attention weight, we notice a considerable gap between the social neurons and the rest of the neurons. The identified social neurons account for more than 90% of the total probability mass. This result is consistent across both MEDAL (a) and MEDAL-ADR (b), and motivate the choice of our deliberately loose threshold (0.05). The other agent used for comparison, MED—, does not have any social neurons because none of the neurons surpasses the threshold.

The linear probing method for identifying a social neuron is as follows:

1. **Data collection** We load a trained agent and run its policy (without further training) for 200 episodes, randomising the goal and the timestep at which dropout occurs. We collect pairs of features, namely the agent's belief state and a corresponding binary label indicating whether or not the expert is present for every timestep in each episode.

2. **Training** We randomise and split the previously collected dataset into a train (70%) and test set (30%). We train a new classification model that predicts whether the expert was present or not at each timestep based on the agent's associated belief state (already trained and frozen). The model is parameterised by an attention map of the same size as the belief (i.e., 512), learning the relative importance of each of its neurons. The attention-weighted belief then passes through a linear layer and is projected onto a 2D space, corresponding to the task's classes. Under this training strategy, we identify social neurons as the maximally attended neurons, those for which the attention weight is higher than a threshold, which we set to 0.05. Supplementary Figure 13 shows that we picked this threshold sensibly: the social neurons selected under this criterion account for more than 90% of the probability mass.

3. **Evaluation** We first check the accuracy of the newly trained classification model on the test set. To causally probe our earlier assumptions, we also make two interventions on the test set: one replaces the identified social neurons with random activations; the other replaces all neurons except the identified social neurons with random activations. Note that we have drawn these random values from a normal distribution resembling that neuron's original activation distribution in the training set. We also report the accuracy on the test set when setting all neurons to random activations drawn from their distributions (random baseline).

4. **Analysis** Finally, we look at the trained attention map and activations of the identified social neurons across episodes.

Having identified social neurons as those maximally weighted in the attention map (Supplementary Figures 14a and 14c), we observe a sharp sign or magnitude change in the activations of the social neurons when the expert drops out. As an example, we plot these across one randomly selected episode for MEDAL (Supplementary Figure 14b) and MEDAL-ADR (Supplementary Figure 14d). shows that the activations of the maximally weighted neurons have meaningfully different magnitudes and opposing signs depending on whether the expert is present or not.

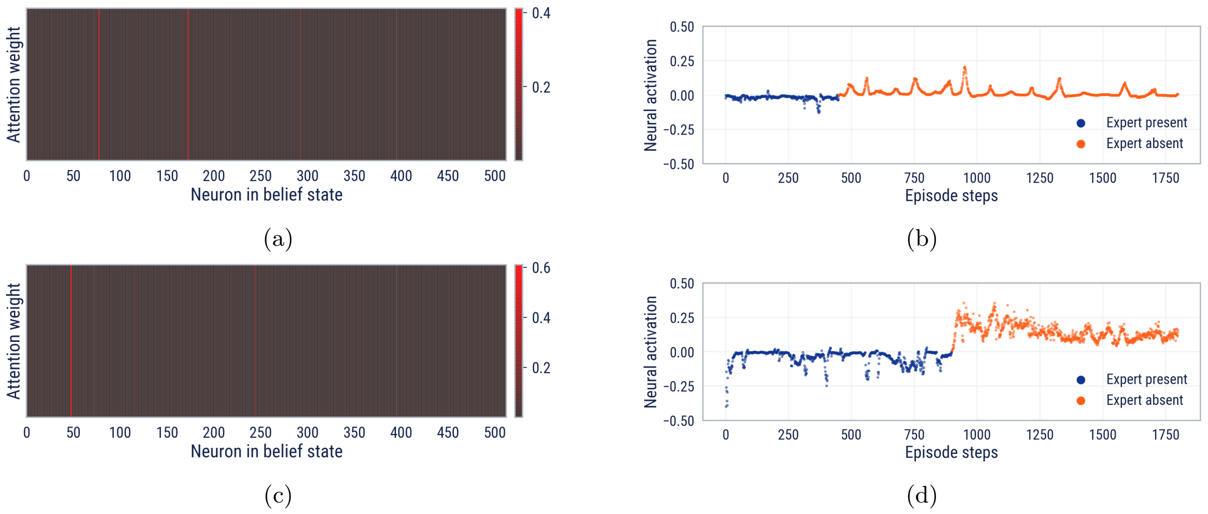

(a) (b) (c) (d)

Supplementary Figure 14: Learned attention map and corresponding social neural activations for MEDAL and MEDAL-ADR from a randomly selected episode. (a) MEDAL's learned attention map. (b) One of MEDAL's social neuron activations. (c) MEDAL-ADR's learned attention map. (d) MEDAL-ADR's social neuron activations.

In Supplementary Figure 15 we provide a snapshot of the activations of 45 neurons in MEDAL-ADR's belief state, where we identify neuron 123 as a "goal neuron". At first glance,

and using the neighbouring neurons for contrast, the goal neuron is characterised by a distinctive periodic variation in its firing pattern across the episode.

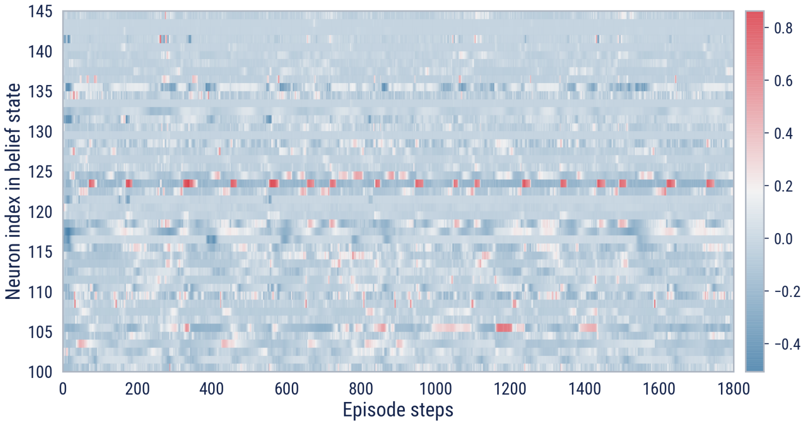

Supplementary Figure 15: Snapshot of neuron activations in MEDAL-ADR's belief state.

E Related work

Our work continues a long line of research about how to generate artificial agents that imitate human behaviour online in rich 3D physical environments, most prominently in the robotics community (see [16] for a review). Much of this work has employed model-based systems to make online learning sample efficient (e.g. [3]), sometimes taking inspiration from prediction mechanisms in the human brain (e.g. [17, 25]). Inspired by this, we ask whether it is possible to use model-free RL to learn an implicit model in the memory of a neural network, which at test-time is capable of online imitation. The answer is "yes". Our approach inherits the scaleability advantages of model-free methods [34], but retains the sample-efficiency of model-based methods at test time. Because our method makes heavy use of domain randomisation, it may even be amenable to use in sim-to-real contexts [55].

Previous works have used model-free reinforcement learning to generate a fixed policy capable of test-time social learning and generalisation to held out tasks. [12] demonstrated that A3C agents [64] were capable of learning to find and follow an expert co-player in a grid-world navigation task, purely on the basis of goal-location reward. Agents trained on a four-room maze generalised in zero-shot to follow in a nine-room maze. When the presence of the expert was dropped out across training, the agent learned to solve the nine-room maze fully independently. However, the authors did not produce a fixed policy capable of reproducing a demonstrated trajectory after expert dropout within a single evaluation episode.

[68] extend the work of [12], demonstrating more rigorously that learning social learning improves generalisation to held-out tasks and achieves better performance than solo baselines on hard exploration problems. Crucial to the success of their agents is a next-step prediction auxiliary loss [45, 78]. The authors introduce the *GoalCycle* environment where task knowledge comprises navigating goals in the correct order, in principle providing parameterised, open-ended navigational complexity. Similarly to previous work, [68] do not generate an agent capable of within-episode recall.

There have been various works leveraging reinforcement learning to generalise to human co-play in coordination tasks, without using human data in the training pipeline. FCP [82] uses a population of self-play agents and their past checkpoints as a training distribution for a focal agent in a grid-world version of Overcooked [22], producing agents that generalise well to human co-play on the same task seen in training. LILA [94] uses multi-agent deep RL to generate an agent capable of inferring a "principal" agent's goal, namely the preference for one

of two object types, and using this information to assist the principal, achieve good performance with human co-players in simple grid-worlds. We take inspiration from these works, but tackle a different problem: high fidelity cultural transmission of long, strategic behaviours.

A line of work in the card game Hanabi [7] is also oriented in this direction, generating zero-shot coordination with human players via various explicit modelling techniques, including by exploiting symmetries [19, 43], learned belief search [44], diversity algorithms [21, 59] and modelling theory of mind [98]. Hanabi is a turn-based game in which all actions are visible and each player has different legal actions, so it is not an appropriate arena in which to study the correspondence problem.

We identify a minimal sufficient "starter kit" of ingredients (MEDAL-ADR) that give rise to a fixed neural network capable of cultural transmission. Many of these ingredients build off prior work. The importance of memory (M) was already identified via ablations in [12], and echoes the presence of recurrent state in meta-RL algorithms [27, 89]. Theoretically, our setting is automatically a POMDP, since the policy of the expert and the details of the task sampling are hidden information. Therefore, we would expect memory to be essential in de-aliasing states via observation of task dynamics and expert behaviour over time [92]. The presence of expert co-players (E) was already ablated in [12]; we provide additional ablations that explore how the reliability of the expert affects the learning of cultural transmission, inspired by the competence dimension of interpersonal perception [30].

Dropout (D) is a well studied mechanism for reducing overfitting in many fields of machine learning [53], and was used across training episodes in [12, 68]. To our knowledge, we are the first to use co-player dropout *within* the course of an episode.

Unsupervised auxiliary losses in reinforcement learning were introduced in [45], and are commonly used to shape representations, promoting more efficient learning, and helping to overcome hard exploration problems. [68] found that a next-step prediction loss was beneficial to promote social learning in a grid-world setting. Reconstruction losses in particular tend to have beneficial effects on generalisation [54]. We use a novel unsupervised auxiliary reconstruction loss focused on the relative position of co-players, which we refer to as an attention loss (AL).

Automatic domain randomisation (ADR) is a technique within the broader field of co-adaptation of agents and environments to yield generalisation. Its precursor, domain randomisation (DR), used uniform sampling of a diverse set of environments and tasks to bridge the sim-to-real gap in robotics [73, 85]. ADR extended DR by automatically adapting the randomisation ranges to agent performance, leading to the zero-shot transfer of a policy for a complex, in-hand manipulation task from simulation to a real-world robot [69]. Other forms of agent-environment co-adaptation include [29, 47, 90]. In the multi-agent setting, agent-agent interactions provide a rich source of autocurricula [6, 26, 56, 81] in which co-operation and competition can scale the task difficulty automatically as agents become more capable.

All of these ingredients have analogues in human and animal cognition. Better working memory (M) is known to correlate with improved fluid intelligence in humans [28] and the ability to solve novel reasoning problems [24], including by imitation [87]. The quality of expert demonstrations (E) is a crucial determinant of the success of cultural transmission in humans, to the extent that humans possess psychological adaptations to enhance quality when multiple models are available, such as prestige bias [39].

The progressive increase in duration of expert dropout (D) mirrors the development of secure attachment in attachment theory [14, 37], where a human child learns to use their caregiver as a safe base for independent behaviour when that caregiver is absent. The success of expert dropout as a means of learning to recall demonstrations across contexts echoes the discovery that interleaving improves inductive learning [52]. The importance of dropout for successful imitation has also been noted in animals [33]: "for the pattern of behaviour initiated by the leader to become part of the behavioural repertoire of the follower, independent of the leader, the pattern of behaviour must come under the control of stimuli not dependent on the presence

of the leader". Humans, along with many animals, have an in-built attentional bias towards biological motion [8, 9], mirroring our attention loss (AL).

Among animals, social learning is known to be preferred over asocial learning in uncertain or varying ecological contexts [2, 79], environment properties we create via domain randomisation (DR). Learning cultural transmission, then, requires that the environment remains consistently in a "Goldilocks zone" of variability, as observed in autocatalytic models [15] and empirical data on climate across human evolution [76, 96]. We achieve this balance by varying the distributional parameters for domain randomisation automatically (A) as a function of the current cultural transmission capability of the agent, keeping the agent in the zone of proximal development [88].

There has been much laboratory work studying cultural transmission in humans, including in situations where humans learn a new behaviour and transmit this behaviour across generations (e.g. [51, 77, 84]). Modelling in such settings is typically challenging, particularly when it comes to the intricate sensorimotor structure of behaviour which characterises much human tool use [35]. On the other hand, there is a long history of interaction between cultural evolution and computational systems. In many such systems, the transmission step is hard-coded directly (e.g. [18, 32, 38, 75]), pre-defining the abstraction of the behaviours that can be represented, and potentially limiting the open-endedness of the method. [93] propose a more "data-driven" transmission system, in which imitation is carried out in third-person by an explicit online learning algorithm. Our work takes this one step further: the ability of cultural transmission is itself learned in a rich sensorimotor environment. Therefore, our methods head towards an even more general and scaleable approach to generating cultural transmission in artificial agents, and perhaps even offers inspiration for modelling.

In RL algorithms, it is common to explore in the space of actions, such as in ϵ-greedy and softmax policies, or when entropy regularisation is used. To overcome local optima in hard exploration problems, authors have proposed exploring in the space of policies via diversity objectives [41], in the space of states via intrinsic motivation [10, 20, 72], in the space of value functions [5, 71] and in the space of neural network weights [31, 74]. Less common is work on exploration in the space of structured behaviours pertinent to the task at hand, for instance in hierarchical RL [80], the kinds of behaviours that humans naturally exchange during cultural transmission. This work helps to fill the gap, solving a hard exploration problem via within-episode cultural transmission, automatically amenable to human interaction. Since this capability is learned, it can be seen as novel form of "meta-exploration" [36, 58].

We may characterise our setting as a form of meta-learning problem: learning to learn from other agents. The trained network must be capable of online adaptation [13] with fixed weights, behaving in a way that is rational in hindsight [65]. The reinforcement learning (RL) training can be thought of as embedding in the neural network's weights the logic for a state-machine capable of (approximately) Bayes-optimal cultural transmission at test time [62]. In our trained agent, we indeed found particular memory neurons that encode a subset of the sufficient statistics required for solving the task [70]. Our chosen task has a periodic structure, generating reliable information that an agent can discover and exploit within an episode. Our agent possesses an LSTM memory [40] and observes its own reward, inspired by the setup in Duan et al. [27], Wang et al. [89]. Apart from these minimal affordances, we do not require any explicit meta-learning algorithms (e.g., [63]) to train our cultural transmission policy.

Supplementary References

[1] A. Abdolmaleki, J. T. Springenberg, Y. Tassa, R. Munos, N. Heess, and M. Riedmiller. Maximum a posteriori policy optimisation. In *International Conference on Learning Representations*, 2018.

[2] L. Aplin. Understanding the multiple factors governing social learning and the diffusion of innovations. *Current opinion in behavioral sciences*, 12:59–65, 2016.

[3] C. Atkeson and S. Schaal. Learning tasks from a single demonstration. In *Proceedings of International Conference on Robotics and Automation*, volume 2, pages 1706–1712 vol.2, 1997. doi: 10.1109/ROBOT.1997.614389.

[4] I. Babuschkin, K. Baumli, A. Bell, S. Bhupatiraju, J. Bruce, P. Buchlovsky, D. Budden, T. Cai, A. Clark, I. Danihelka, C. Fantacci, J. Godwin, C. Jones, T. Hennigan, M. Hessel, S. Kapturowski, T. Keck, I. Kemaev, M. King, L. Martens, V. Mikulik, T. Norman, J. Quan, G. Papamakarios, R. Ring, F. Ruiz, A. Sanchez, R. Schneider, E. Sezener, S. Spencer, S. Srinivasan, W. Stokowiec, and F. Viola. The DeepMind JAX Ecosystem, 2020. URL http://github.com/deepmind.

[5] A. P. Badia, P. Sprechmann, A. Vitvitskyi, D. Guo, B. Piot, S. Kapturowski, O. Tieleman, M. Arjovsky, A. Pritzel, A. Bolt, and C. Blundell. Never give up: Learning directed exploration strategies, 2020.

[6] B. Baker, I. Kanitscheider, T. Markov, Y. Wu, G. Powell, B. McGrew, and I. Mordatch. Emergent tool use from multi-agent autocurricula. In *International Conference on Learning Representations*, 2020.

[7] N. Bard, J. N. Foerster, S. Chandar, N. Burch, M. Lanctot, H. F. Song, E. Parisotto, V. Dumoulin, S. Moitra, E. Hughes, et al. The hanabi challenge: A new frontier for ai research. *Artificial Intelligence*, 280:103216, 2020.

[8] L. Bardi, L. Regolin, and F. Simion. Biological motion preference in humans at birth: Role of dynamic and configural properties. *Developmental science*, 14(2):353–359, 2011.

[9] L. Bardi, L. Regolin, and F. Simion. The first time ever i saw your feet: Inversion effect in newborns' sensitivity to biological motion. *Developmental psychology*, 50(4):986, 2014.

[10] M. Bellemare, S. Srinivasan, G. Ostrovski, T. Schaul, D. Saxton, and R. Munos. Unifying count-based exploration and intrinsic motivation. *Advances in neural information processing systems*, 29, 2016.

[11] J. Bevins. Libnoise. 2003.

[12] D. Borsa, N. Heess, B. Piot, S. Liu, L. Hasenclever, R. Munos, and O. Pietquin. Observational learning by reinforcement learning. In *Proceedings of the 18th International Conference on Autonomous Agents and MultiAgent Systems*, pages 1117–1124, 2019.

[13] L. Bottou. *On-Line Learning and Stochastic Approximations*, page 9–42. Cambridge University Press, USA, 1999. ISBN 0521652634.

[14] J. Bowlby. The nature of the child's tie to his mother. *International journal of psychoanalysis*, 39:350–373, 1958.

[15] R. Boyd and P. J. Richerson. *Culture and the evolutionary process*. University of Chicago press, 1988.

[16] C. Breazeal and B. Scassellati. Robots that imitate humans. *Trends in Cognitive Sciences*, 6 (11):481–487, 2002. ISSN 1364-6613. doi: https://doi.org/10.1016/S1364-6613(02)02016-8. URL https://www.sciencedirect.com/science/article/pii/S1364661302020168.

[17] C. Breazeal, D. Buchsbaum, J. Gray, D. Gatenby, and B. Blumberg. Learning from and about others: Towards using imitation to bootstrap the social understanding of others by robots. *Artificial Life*, 11(1-2):31–62, 2005. doi: 10.1162/1064546053278955.

[18] N. Bredeche and N. Fontbonne. Social learning in swarm robotics. *Philosophical Transactions of the Royal Society B*, 377(1843):20200309, 2022.

[19] K. Bullard, D. Kiela, F. Meier, J. Pineau, and J. Foerster. Quasi-equivalence discovery for zero-shot emergent communication. *arXiv preprint arXiv:2103.08067*, 2021.

[20] Y. Burda, H. Edwards, A. Storkey, and O. Klimov. Exploration by random network distillation. In *Seventh International Conference on Learning Representations*, pages 1–17, 2019.

[21] R. Canaan, X. Gao, J. Togelius, A. Nealen, and S. Menzel. Generating and adapting to diverse ad-hoc partners in hanabi. *IEEE Transactions on Games*, pages 1–1, 2022. doi: 10.1109/TG.2022.3169168.

[22] M. Carroll, R. Shah, M. K. Ho, T. Griffiths, S. Seshia, P. Abbeel, and A. Dragan. On the utility of learning about humans for human-ai coordination. *Advances in Neural Information Processing Systems*, 32:5174–5185, 2019.

[23] A. Cassirer, G. Barth-Maron, E. Brevdo, S. Ramos, T. Boyd, T. Sottiaux, and M. Kroiss. Reverb: A framework for experience replay, 2021.

[24] R. B. Cattell. Theory of fluid and crystallized intelligence: A critical experiment. *Journal of educational psychology*, 54(1):1, 1963.

[25] Y. Demiris and M. Johnson. Distributed, predictive perception of actions: a biologically inspired robotics architecture for imitation and learning. *Connection Science*, 15 (4):231–243, 2003. doi: 10.1080/0954009031000165512 9. URL https://doi.org/10.1080/09540090310001655129.

[26] M. Dennis, N. Jaques, E. Vinitsky, A. Bayen, S. Russell, A. Critch, and S. Levine. Emergent complexity and zero-shot transfer via unsupervised environment design. *Advances in neural information processing systems*, 33:13049–13061, 2020.

[27] Y. Duan, J. Schulman, X. Chen, P. L. Bartlett, I. Sutskever, and P. Abbeel. Rl^2: Fast reinforcement learning via slow reinforcement learning. *arXiv preprint arXiv:1611.02779*, 2016.

[28] J. Duncan, M. Schramm, R. Thompson, and I. Dumontheil. Task rules, working memory, and fluid intelligence. *Psychonomic bulletin & review*, 19(5):864–870, 2012.

[29] R. Everett, A. Cobb, A. Markham, and S. Roberts. Optimising worlds to evaluate and influence reinforcement learning agents. In *Proceedings of the 18th International Conference on Autonomous Agents and MultiAgent Systems*, pages 1943–1945, 2019.

[30] S. T. Fiske, A. J. Cuddy, P. Glick, and J. Xu. A model of (often mixed) stereotype content: competence and warmth respectively follow from perceived status and competition. *Journal of personality and social psychology*, 82(6):878, 2002.

[31] M. Fortunato, M. G. Azar, B. Piot, J. Menick, M. Hessel, I. Osband, A. Graves, V. Mnih, R. Munos, D. Hassabis, et al. Noisy networks for exploration. In *International Conference on Learning Representations*, 2018.

[32] L. Gabora. Meme and variations: a computer model of cultural evolution. In *In Nadel, L. and Stein, D.(Eds.), 1993 Lectures in Complex Systems, Addison-Wesley.* 1995.

[33] B. G. Galef Jr. Imitation in animals: History, definition, and interpretation of data from the psychological laboratory. In T. R. Zentall and B. G. Galef Jr., editors, *Social learning: psychological and biological perspectives*, pages 3–28. Lawrence Erlbaum Associates, Inc., 1988.

[34] H. Geffner. Model-free, model-based, and general intelligence. In *Proceedings of the 27th International Joint Conference on Artificial Intelligence*, pages 10–17, 2018.

[35] F. Guerin, N. Krüger, and D. Kraft. A survey of the ontogeny of tool use: From sensorimotor experience to planning. *Autonomous Mental Development, IEEE Transactions on*, 03 2013. doi: 10.1109/TAMD.2012.2209879.

[36] A. Gupta, R. Mendonca, Y. Liu, P. Abbeel, and S. Levine. Meta-reinforcement learning of structured exploration strategies. In *Proceedings of the 32nd International Conference on Neural Information Processing Systems*, NIPS'18, page 5307–5316, Red Hook, NY, USA, 2018. Curran Associates Inc.

[37] H. F. Harlow. The nature of love. *American psychologist*, 13(12):673, 1958.

[38] E. Hart and L. K. Le Goff. Artificial evolution of robot bodies and control: on the interaction between evolution, learning and culture. *Philosophical Transactions of the Royal Society B*, 377(1843):20210117, 2022.

[39] J. Henrich and F. J. Gil-White. The evolution of prestige: Freely conferred deference as a mechanism for enhancing the benefits of cultural transmission. *Evolution and human behavior*, 22(3):165–196, 2001.

[40] S. Hochreiter and J. Schmidhuber. Long short-term memory. *Neural computation*, 9(8): 1735–1780, 1997.

[41] Z.-W. Hong, T.-Y. Shann, S.-Y. Su, Y.-H. Chang, T.-J. Fu, and C.-Y. Lee. Diversity-driven exploration strategy for deep reinforcement learning. In *Proceedings of the 32nd International Conference on Neural Information Processing Systems*, pages 10510–10521, 2018.

[42] R. A. Howard. *Dynamic Programming and Markov Processes.* MIT Press, Cambridge, MA, 1960.

[43] H. Hu, A. Lerer, A. Peysakhovich, and J. Foerster. "other-play" for zero-shot coordination. In *International Conference on Machine Learning*, pages 4399–4410. PMLR, 2020.

[44] H. Hu, A. Lerer, N. Brown, and J. Foerster. Learned belief search: Efficiently improving policies in partially observable settings. *arXiv preprint arXiv:2106.09086*, 2021.

[45] M. Jaderberg, V. Mnih, W. M. Czarnecki, T. Schaul, J. Z. Leibo, D. Silver, and K. Kavukcuoglu. Reinforcement learning with unsupervised auxiliary tasks. In *International Conference on Learning Representations*, 2017.

[46] M. Jaderberg, W. M. Czarnecki, I. Dunning, L. Marris, G. Lever, A. G. Castaneda, C. Beattie, N. C. Rabinowitz, A. S. Morcos, A. Ruderman, et al. Human-level performance in 3d multiplayer games with population-based reinforcement learning. *Science*, 364(6443):859–865, 2019.

[47] M. Jiang, M. Dennis, J. Parker-Holder, J. Foerster, E. Grefenstette, and T. Rocktäschel. Replay-guided adversarial environment design. *Advances in Neural Information Processing Systems*, 34:1884–1897, 2021.

[48] A. Juliani, V.-P. Berges, E. Teng, A. Cohen, J. Harper, C. Elion, C. Goy, Y. Gao, H. Henry, M. Mattar, et al. Unity: A general platform for intelligent agents. *arXiv preprint arXiv:1809.02627*, 2018.

[49] M. Kearns and S. Singh. Near-optimal reinforcement learning in polynomial time. *Machine learning*, 49(2):209–232, 2002.

[50] D. P. Kingma and J. Ba. Adam: A method for stochastic optimization. *CoRR*, abs/1412.6980, 2015.

[51] S. Kirby, H. Cornish, and K. Smith. Cumulative cultural evolution in the laboratory: An experimental approach to the origins of structure in human language. *Proceedings of the National Academy of Sciences*, 105(31):10681–10686, 2008.

[52] N. Kornell and R. A. Bjork. Learning concepts and categories: Is spacing the "enemy of induction"? *Psychological science*, 19(6):585–592, 2008.

[53] A. Labach, H. Salehinejad, and S. Valaee. Survey of dropout methods for deep neural networks. *arXiv preprint arXiv:1904.13310*, 2019.

[54] L. Le, A. Patterson, and M. White. Supervised autoencoders: Improving generalization performance with unsupervised regularizers. *Advances in neural information processing systems*, 31:107–117, 2018.

[55] J. Lee, J. Hwangbo, L. Wellhausen, V. Koltun, and M. Hutter. Learning quadrupedal locomotion over challenging terrain. *Science robotics*, 5(47):eabc5986, 2020.

[56] J. Z. Leibo, E. Hughes, M. Lanctot, and T. Graepel. Autocurricula and the emergence of innovation from social interaction: A manifesto for multi-agent intelligence research, 2019.

[57] M. L. Littman. Markov games as a framework for multi-agent reinforcement learning. In *Machine learning proceedings 1994*, pages 157–163. Elsevier, 1994.

[58] E. Z. Liu, A. Raghunathan, P. Liang, and C. Finn. Decoupling exploration and exploitation for meta-reinforcement learning without sacrifices. In *International conference on machine learning*, pages 6925–6935. PMLR, 2021.

[59] A. Lupu, B. Cui, H. Hu, and J. Foerster. Trajectory diversity for zero-shot coordination. In *International Conference on Machine Learning*, pages 7204–7213. PMLR, 2021.

[60] F. B. Malavazi, R. Guyonneau, J.-B. Fasquel, S. Lagrange, and F. Mercier. Lidar-only based navigation algorithm for an autonomous agricultural robot. *Computers and Electronics in Agriculture*, 154:71–79, 2018.

[61] K. R. McKee, J. Z. Leibo, C. Beattie, and R. Everett. Quantifying the effects of environment and population diversity in multi-agent reinforcement learning. *Autonomous Agents and Multi-Agent Systems*, 36(1):21, 2022.

[62] V. Mikulik, G. Delétang, T. McGrath, T. Genewein, M. Martic, S. Legg, and P. A. Ortega. Meta-trained agents implement bayes-optimal agents. In *Proceedings of the 34th International Conference on Neural Information Processing Systems*, pages 18691–18703, 2020.

[63] E. Mitchell, R. Rafailov, X. B. Peng, S. Levine, and C. Finn. Offline meta-reinforcement learning with advantage weighting. In *International Conference on Machine Learning*, pages 7780–7791. PMLR, 2021.

[64] V. Mnih, A. P. Badia, M. Mirza, A. Graves, T. Lillicrap, T. Harley, D. Silver, and K. Kavukcuoglu. Asynchronous methods for deep reinforcement learning. In *International conference on machine learning*, pages 1928–1937. PMLR, 2016.

[65] D. Morrill, R. D'Orazio, R. Sarfati, M. Lanctot, J. R. Wright, A. R. Greenwald, and M. Bowling. Hindsight and sequential rationality of correlated play. In *Proceedings of the AAAI Conference on Artificial Intelligence*, volume 35, pages 5584–5594, 2021.

[66] A. Muldal, Y. Doron, J. Aslanides, T. Harley, T. Ward, and S. Liu. dm_env: A python interface for reinforcement learning environments, 2019. URL http://github.com/deepmind/dm_env.

[67] R. Munos, T. Stepleton, A. Harutyunyan, and M. Bellemare. Safe and efficient off-policy reinforcement learning. *Advances in neural information processing systems*, 29, 2016.

[68] K. K. Ndousse, D. Eck, S. Levine, and N. Jaques. Emergent social learning via multi-agent reinforcement learning. In *International Conference on Machine Learning*, pages 7991–8004. PMLR, 2021.

[69] OpenAI, I. Akkaya, M. Andrychowicz, M. Chociej, M. Litwin, B. McGrew, A. Petron, A. Paino, M. Plappert, G. Powell, R. Ribas, J. Schneider, N. Tezak, J. Tworek, P. Welinder, L. Weng, Q. Yuan, W. Zaremba, and L. Zhang. Solving rubik's cube with a robot hand. *arXiv preprint arXiv:1910.07113*, 2019.

[70] P. A. Ortega, J. X. Wang, M. Rowland, T. Genewein, Z. Kurth-Nelson, R. Pascanu, N. Heess, J. Veness, A. Pritzel, P. Sprechmann, et al. Meta-learning of sequential strategies. *arXiv preprint arXiv:1905.03030*, 2019.

[71] I. Osband, B. Van Roy, D. J. Russo, Z. Wen, et al. Deep exploration via randomized value functions. *J. Mach. Learn. Res.*, 20(124):1–62, 2019.

[72] D. Pathak, P. Agrawal, A. A. Efros, and T. Darrell. Curiosity-driven exploration by self-supervised prediction. In *International conference on machine learning*, pages 2778–2787. PMLR, 2017.

[73] X. B. Peng, M. Andrychowicz, W. Zaremba, and P. Abbeel. Sim-to-real transfer of robotic control with dynamics randomization. In *2018 IEEE international conference on robotics and automation (ICRA)*, pages 3803–3810. IEEE, 2018.

[74] M. Plappert, R. Houthooft, P. Dhariwal, S. Sidor, R. Y. Chen, X. Chen, T. Asfour, P. Abbeel, and M. Andrychowicz. Parameter space noise for exploration. In *International Conference on Learning Representations*, 2018.

[75] L. Rendell, R. Boyd, D. Cownden, M. Enquist, K. Eriksson, M. W. Feldman, L. Fogarty, S. Ghirlanda, T. Lillicrap, and K. N. Laland. Why copy others? insights from the social learning strategies tournament. *Science*, 328(5975):208–213, 2010.

[76] P. J. Richerson, R. L. Bettinger, and R. Boyd. Evolution on a restless planet: Were environmental variability and environmental change major drivers of human evolution. *Handbook of evolution*, 2:223–242, 2005.

[77] C. Saldana, J. Fagot, S. Kirby, K. Smith, and N. Claidière. Cumulative cultural evolution in a non-copying task in children and guinea baboons. In *41st Annual Meeting of the Cognitive Science Society*, pages 1001–1007. Cognitive Science Society, 2019.

[78] E. Shelhamer, P. Mahmoudieh, M. Argus, and T. Darrell. Loss is its own reward: Self-supervision for reinforcement learning. *arXiv preprint arXiv:1612.07307*, 2016.

[79] M. Smolla, S. Alem, L. Chittka, and S. Shultz. Copy-when-uncertain: bumblebees rely on social information when rewards are highly variable. *Biology letters*, 12(6):20160188, 2016.

[80] L. Steccanella, S. Totaro, D. Allonsius, and A. Jonsson. Hierarchical reinforcement learning for efficient exploration and transfer. *arXiv preprint arXiv:2011.06335*, 2020.

[81] A. Stooke, A. Mahajan, C. Barros, C. Deck, J. Bauer, J. Sygnowski, M. Trebacz, M. Jaderberg, M. Mathieu, N. McAleese, N. Bradley-Schmieg, N. Wong, N. Porcel, R. Raileanu, S. Hughes-Fitt, V. Dalibard, and W. M. Czarnecki. Open-ended learning leads to generally capable agents. *arXiv preprint arXiv:2107.12808*, 2021.

[82] D. Strouse, K. R. McKee, M. Botvinick, E. Hughes, and R. Everett. Collaborating with humans without human data. In *Neural Information Processing Systems (NeurIPS)*, 2021.

[83] R. S. Sutton and A. G. Barto. *Reinforcement learning: An introduction*. MIT press, 2018.

[84] M. H. Tessler, P. A. Tsividis, J. Madeano, B. Harper, and J. B. Tenenbaum. Growing knowledge culturally across generations to solve novel, complex tasks. *CoRR*, abs/2107.13377, 2021. URL https://arxiv.org/abs/2107.13377.

[85] J. Tobin, R. Fong, A. Ray, J. Schneider, W. Zaremba, and P. Abbeel. Domain randomization for transferring deep neural networks from simulation to the real world. In *2017 IEEE/RSJ International Conference on Intelligent Robots and Systems (IROS)*, page 23–30. IEEE Press, 2017. doi: 10.1109/IROS.2017.8202133. URL https://doi.org/10.1109/IROS.2017.8202133.

[86] W. van Toll, A. Iv, and R. Geraerts. A navigation mesh for dynamic environments. *Computer Animation and Virtual Worlds*, 23:535–546, 11 2012. doi: 10.1002/cav.1468.

[87] A. Vostroknutov, L. Polonio, and G. Coricelli. The role of intelligence in social learning. *Scientific reports*, 8(1):1–10, 2018.

[88] L. S. Vygotsky. *Mind in society: The development of higher psychological processes*. Harvard university press, 1980.

[89] J. X. Wang, Z. Kurth-Nelson, D. Tirumala, H. Soyer, J. Z. Leibo, R. Munos, C. Blundell, D. Kumaran, and M. Botvinick. Learning to reinforcement learn. *arXiv preprint arXiv:1611.05763*, 2016.

[90] R. Wang, J. Lehman, J. Clune, and K. O. Stanley. Poet: open-ended coevolution of environments and their optimized solutions. In *Proceedings of the Genetic and Evolutionary Computation Conference*, pages 142–151, 2019.

[91] T. Ward, A. Bolt, N. Hemmings, S. Carter, M. Sanchez, R. Barreira, S. Noury, K. Anderson, J. Lemmon, J. Coe, P. Trochim, T. Handley, and A. Bolton. Using unity to help solve intelligence. *arXiv preprint arXiv:2011.09294*, 2020.

[92] D. Wierstra, A. Foerster, J. Peters, and J. Schmidhuber. Solving deep memory pomdps with recurrent policy gradients. In *International conference on artificial neural networks*, pages 697–706. Springer, 2007.

[93] A. F. Winfield and S. Blackmore. Experiments in artificial culture: from noisy imitation to storytelling robots. *Philosophical Transactions of the Royal Society B*, 377(1843):20200323, 2022.

[94] M. Woodward, C. Finn, and K. Hausman. Learning to interactively learn and assist. In *Proceedings of the AAAI conference on artificial intelligence*, volume 34, pages 2535–2543, 2020.

[95] F. Yang, G. Barth-Maron, P. Stańczyk, M. Hoffman, S. Liu, M. Kroiss, A. Pope, and A. Rrustemi. Launchpad: A programming model for distributed machine learning research. *arXiv preprint arXiv:2106.04516*, 2021. URL https://arxiv.org/abs/2106.04516.

[96] J. Zachos, M. Pagani, L. Sloan, E. Thomas, and K. Billups. Trends, rhythms, and aberrations in global climate 65 ma to present. *Science*, 292(5517):686–693, 2001.

[97] C. Zhang, O. Vinyals, R. Munos, and S. Bengio. A study on overfitting in deep reinforcement learning. *arXiv preprint arXiv:1804.06893*, 2018.

[98] H. Zhu, G. Neubig, and Y. Bisk. Few-shot language coordination by modeling theory of mind. In *International Conference on Machine Learning*, pages 12901–12911. PMLR, 2021.
