# Supplementary material for: Learning few-shot imitation as cultural transmission
Source: Nat Commun. 2023 Nov 28;14:7536. doi: 10.1038/s41467-023-42875-2 (PMC10684502; doi:10.1038/s41467-023-42875-2)
Supplement: Supplementary file 3 — Reporting Summary [file 41467_2023_42875_MOESM3_ESM.pdf]

nature portfolio

Corresponding author(s): Edward Hughes

Last updated by author(s): Sep 14, 2023

Reporting Summary

Nature Portfolio wishes to improve the reproducibility of the work that we publish. This form provides structure for consistency and transparency in reporting. For further information on Nature Portfolio policies, see our Editorial Policies and the Editorial Policy Checklist.

Statistics

For all statistical analyses, confirm that the following items are present in the figure legend, table legend, main text, or Methods section.

n/a	Confirmed	
☐	☒	The exact sample size (*n*) for each experimental group/condition, given as a discrete number and unit of measurement
☒	☐	A statement on whether measurements were taken from distinct samples or whether the same sample was measured repeatedly
☒	☐	The statistical test(s) used AND whether they are one- or two-sided<br>*Only common tests should be described solely by name; describe more complex techniques in the Methods section.*
☒	☐	A description of all covariates tested
☒	☐	A description of any assumptions or corrections, such as tests of normality and adjustment for multiple comparisons
☐	☒	A full description of the statistical parameters including central tendency (e.g. means) or other basic estimates (e.g. regression coefficient) AND variation (e.g. standard deviation) or associated estimates of uncertainty (e.g. confidence intervals)
☒	☐	For null hypothesis testing, the test statistic (e.g. *F*, *t*, *r*) with confidence intervals, effect sizes, degrees of freedom and *P* value noted<br>*Give P values as exact values whenever suitable.*
☒	☐	For Bayesian analysis, information on the choice of priors and Markov chain Monte Carlo settings
☒	☐	For hierarchical and complex designs, identification of the appropriate level for tests and full reporting of outcomes
☒	☐	Estimates of effect sizes (e.g. Cohen's *d*, Pearson's *r*), indicating how they were calculated

Our web collection on statistics for biologists contains articles on many of the points above.

Software and code

Policy information about availability of computer code

Data collection	We are unable to release the code for this work as it was developed in a proprietary context. We are happy to answer specific questions concerning re-implementation: please contact reverett@deepmind.com. An open-source implementation of the MPO algorithm is available at https://github.com/deepmind/acme/tree/master/acme/agents/jax/mpo. An open-source implementation of the 2D variant of GoalCycle is available at https://github.com/kandouss/marlgrid/blob/master/marlgrid/envs/goalcycle.py.
Data analysis	For data analysis we used the following freely available packages: numpy v1.25.2, pandas v1.5.3, matplotlib v3.6.1, seaborn v0.12.2, scipy v1.9.3.

For manuscripts utilizing custom algorithms or software that are central to the research but not yet described in published literature, software must be made available to editors and reviewers. We strongly encourage code deposition in a community repository (e.g. GitHub). See the Nature Portfolio guidelines for submitting code & software for further information.

Data

Policy information about availability of data

All manuscripts must include a data availability statement. This statement should provide the following information, where applicable:

- Accession codes, unique identifiers, or web links for publicly available datasets
- A description of any restrictions on data availability
- For clinical datasets or third party data, please ensure that the statement adheres to our policy

Data for this study was generated via a Unity-based simulation with no additional external data sources. Source data for main text figures are provided with this paper, excluding Figures 6 and 8c, for which analysis data was generated on-the-fly and not logged. Video footage is available on a publicly accessible website at https://sites.google.com/view/dm-cgi.

Research involving human participants, their data, or biological material

Policy information about studies with human participants or human data. See also policy information about sex, gender (identity/presentation), and sexual orientation and race, ethnicity and racism.

Reporting on sex and gender	N/A
Reporting on race, ethnicity, or other socially relevant groupings	N/A
Population characteristics	N/A
Recruitment	N/A
Ethics oversight	N/A

Note that full information on the approval of the study protocol must also be provided in the manuscript.

Field-specific reporting

Please select the one below that is the best fit for your research. If you are not sure, read the appropriate sections before making your selection.

☐ Life sciences ☒ Behavioural & social sciences ☐ Ecological, evolutionary & environmental sciences

For a reference copy of the document with all sections, see nature.com/documents/nr-reporting-summary-flat.pdf

Behavioural & social sciences study design

All studies must disclose on these points even when the disclosure is negative.

Study description	This study is a mixed-methods study, involving both qualitative and quantitative analysis of trained artificial intelligence (AI) agents.
Research sample	The dataset on which our AI agents were trained was generated entirely by a Unity simulation, with no additional external data. To qualitatively demonstrate cultural transmission from a human to an agent, we recorded human trajectories in the simulation generated from gameplay by a member of the authorship team.
Sampling strategy	For our statistical analysis of agent train and test performance we ran as many seeds as was feasible under compute and time constraints, so as to get the best possible understanding of the performance distribution. These seeds were sampled uniformly at random.
Data collection	Please see "research sample" above, which describes the source of all data used in this work.
Timing	This is not applicable to our study, since data for training agents was generated "on-the-fly" and trajectories for human evaluation were collected in a single gameplay session.
Data exclusions	No data was excluded from the analyses.
Non-participation	No human participants were involved in the study, beyond the trajectories of human gameplay recorded by a member of the research team.
Randomization	Randomization was not required for this study since the object of study / ablation are various types of AI agents, the differences

Randomization	between which are known and controlled by the researchers.

Reporting for specific materials, systems and methods

We require information from authors about some types of materials, experimental systems and methods used in many studies. Here, indicate whether each material, system or method listed is relevant to your study. If you are not sure if a list item applies to your research, read the appropriate section before selecting a response.

Materials & experimental systems

n/a	Involved in the study
☒	☐ Antibodies
☒	☐ Eukaryotic cell lines
☒	☐ Palaeontology and archaeology
☒	☐ Animals and other organisms
☒	☐ Clinical data
☒	☐ Dual use research of concern
☒	☐ Plants

Methods

n/a	Involved in the study
☒	☐ ChIP-seq
☒	☐ Flow cytometry
☒	☐ MRI-based neuroimaging
