# Supplementary material for: Learning few-shot imitation as cultural transmission
Source: Nat Commun. 2023 Nov 28;14:7536. doi: 10.1038/s41467-023-42875-2 (PMC10684502; doi:10.1038/s41467-023-42875-2)
Supplement: Supplementary file 4 — Description of Additional Supplementary Files Document [file 41467_2023_42875_MOESM4_ESM.pdf]

Description of Additional Supplementary Files

Supplementary Movies

Supplementary Movie 1: Training Phase 1 (Initial Exploration, 8.6 bn Steps)

Supplementary Movie 2: Training Phase 2 (Following, 15.9bn Steps)

Supplementary Movie 3: Training Phase 3 (Memorization, 18.2bn Steps)

Supplementary Movie 4: Training Phase 4 (Independence, 26.7bn Steps)

Supplementary Movie 5: Solitary Agent Behaviour (M----)

Supplementary Movie 6: Effects of ADR (Horizontal Obstacles)

Supplementary Movie 7: Effects of ADR (Larger World)

Supplementary Movie 8: Effects of ADR (Vertical Obstacles)

Supplementary Movie 9: Effects of ADR (Bumpy Terrain)

Supplementary Movie 10: Recall Analysis (3600 Step Episode)

Supplementary Movie 11: Trajectory Plot (Bot Absent)

Supplementary Movie 12: Trajectory Plot (Bot Dropout)

Supplementary Movie 13: Trajectory Plot (Wrong Demonstration)

Supplementary Movie 14: World Space Generalisation (Empty)

Supplementary Movie 15: World Space Generalisation (Terrain)

Supplementary Movie 16: World Space Generalisation (Obstacles)

Supplementary Movie 17: World Space Generalisation (Terrain and Obstacles)

Supplementary Movie 18: Task Space Generalisation (4 Spheres, 1 Crossing)

Supplementary Movie 19: Task Space Generalisation (5 Spheres, 2 Crossings)

Supplementary Movie 20: Task Space Generalisation (6 Spheres, 4 Crossings)

Supplementary Movie 21: Probe Task (Empty World, 4-Goal Game, Full Expert Demonstration)

Supplementary Movie 22: Probe Task (Empty World, 4-Goal Game, Expert Drops Out Halfway)

Supplementary Movie 23: Probe Task (Empty World, 4-Goal Game, No Expert Demonstration)

Supplementary Movie 24: Probe Task (Empty World, 5-Goal Game, Full Expert Demonstration)

Supplementary Movie 25: Probe Task (Empty World, 5-Goal Game, Expert Drops Out Halfway)

Supplementary Movie 26: Probe Task (Empty World, 5-Goal Game, No Expert Demonstration)

Supplementary Movie 27: Probe Task (Complex World, 5-Goal Game, Full Expert

Demonstration)

Supplementary Movie 28: Probe Task (Complex World, 5-Goal Game, Expert Drops Out Halfway)

Supplementary Movie 29: Probe Task (Complex World, 5-Goal Game, No Expert Demonstration)

Supplementary Movie 30: The LIDAR Sensor

Supplementary Movie 31: MEDAL-ADR Agent Behaviour
